# Activation of the aryl hydrocarbon receptor improves allergen-specific immunotherapy of murine allergic airway inflammation: a novel adjuvant option?

**DOI:** 10.3389/fimmu.2024.1397072

**Published:** 2024-06-10

**Authors:** Sonja Heine, Francesca Alessandrini, Johannes Grosch, Carina Graß, Alexander Heldner, Benjamin Schnautz, Johanna Grosch, Jeroen Buters, Benjamin O. Slusarenko, Daniel Krappmann, Francesca Fallarino, Caspar Ohnmacht, Carsten B. Schmidt-Weber, Simon Blank

**Affiliations:** ^1^ Center of Allergy and Environment (ZAUM), Technical University of Munich, School of Medicine and Health & Helmholtz Munich, German Research Center for Environmental Health, Member of the German Center of Lung Research (DZL), Member of the Immunology and Inflammation Initiative of the Helmholtz Association, Munich, Germany; ^2^ Research Unit Signaling and Translation, Group Signaling and Immunity, Molecular Targets and Therapeutic Center, Helmholtz Munich, German Research Center for Environmental Health, Munich, Germany; ^3^ Department of Medicine and Surgery, University of Perugia, Perugia, Italy

**Keywords:** allergen-specific immunotherapy, AhR knockout mice, adjuvant, aryl hydrocarbon receptor, allergic airway inflammation, immunomodulation, 10-Cl-BBQ

## Abstract

**Background:**

Allergen-specific immunotherapy (AIT) is able to restore immune tolerance to allergens in allergic patients. However, some patients do not or only poorly respond to current treatment protocols. Therefore, there is a need for deeper mechanistic insights and further improvement of treatment strategies. The relevance of the aryl hydrocarbon receptor (AhR), a ligand-dependent transcription factor, has been investigated in several inflammatory diseases, including allergic asthma. However, its potential role in AIT still needs to be addressed.

**Methods:**

A murine model of AIT in ovalbumin-induced allergic airway inflammation was performed in AhR-deficient (AhR^-/-^) and wild-type mice. Furthermore, AIT was combined with the application of the high-affinity AhR agonist 10-chloro-7H-benzimidazo[2,1-a]benzo[de]iso-quinolin-7-one (10-Cl-BBQ) as an adjuvant to investigate the effects of AhR activation on therapeutic outcome.

**Results:**

Although AhR^-/-^ mice suffer stronger allergic responses than wild-type mice, experimental AIT is comparably effective in both. Nevertheless, combining AIT with the administration of 10-Cl-BBQ improved therapeutic effects by an AhR-dependent mechanism, resulting in decreased cell counts in the bronchoalveolar fluid, decreased pulmonary Th2 and Th17 cell levels, and lower sIgE levels.

**Conclusion:**

This study demonstrates that the success of AIT is not dependent on the AhR. However, targeting the AhR during AIT can help to dampen inflammation and improve tolerogenic vaccination. Therefore, AhR ligands might represent promising candidates as immunomodulators to enhance the efficacy of AIT.

## 1 Introduction

Allergen-specific immunotherapy (AIT) is currently the only available causative, disease-modifying treatment option for allergic diseases (1–3) that is able to modulate the immune response and restore immune tolerance towards allergens (4–6). However, its efficacy strongly varies in different patients and types of allergies (7). To improve AIT, a better understanding of its underlying mechanisms, along with the investigation of novel therapy-supporting adjuvants and immunomodulators, is recommended.

The aryl hydrocarbon receptor (AhR) is a ligand-activated transcription factor involved in several physiological processes (8, 9). It can be activated by various exogenous and endogenous ligands provided by dietary intake, host metabolism, pollutants, and microbiota (10). The most famous and best-characterized exogenous high-affinity ligands are polycyclic aromatic hydrocarbons, polychlorinated biphenyls, and halogenated dioxins. In addition, several chemicals with striking structural diversity can induce the AhR (11). Indoles generated endogenously by bacterial metabolism of tryptophan or taken up from exogenous sources by dietary intake are another critical group of AhR ligands (12). Structural diversity and varying affinity of various possible ligands can lead to different effects of AhR activation (13). However, the induction of cytochrome P450 family 1 subfamily 1 member 1 (CYP1A1) is commonly used to identify AhR-active compounds (14–17).

Although historically known for its function as an environmental sensor, research over the past years has also revealed an essential role of the AhR in various immune cells (18–20). AhR ligands induce different cellular and epigenetic processes to attenuate inflammation, making the AhR an attractive therapeutic target (21). Mechanisms through which the AhR can attenuate inflammation include thymic atrophy (22), apoptosis (23), induction of regulatory T cells (Tregs) (24) and myeloid-derived suppressor cells (25), cytokine suppression (26), and epigenetic changes (27). Activation of the AhR has shown promising results in preventing and treating inflammatory diseases, e.g., preventing colitis and associated dysbiosis in a murine model (28) and suppressing experimental multiple sclerosis (29). The AhR can directly regulate the differentiation of T helper (Th) 17 and Tregs, thereby attenuating the development and severity of autoimmune diseases (30, 31). Additionally, the AhR can control the differentiation and function of dendritic cells (DCs) (32, 33). For the inflammatory skin diseases atopic dermatitis and psoriasis, a topical AhR agonist, Tapinarof, is already on the market (21). It has been shown to restore skin homeostasis by decreasing the production of pro-inflammatory cytokines, reducing oxidative stress, and increasing the expression of epithelial barrier genes (34).

In allergic diseases, targeting the AhR with respective ligands has been shown to inhibit Th2 cell differentiation, reduce the Th2 response, and suppress disease progression in a murine model of atopic asthma (35–37). In contrast, the role of the AhR in AIT has not been evaluated so far. Therefore, this study addresses whether the involvement of the AhR is essential for the success of AIT by applying a previously described experimental AIT model of ovalbumin (OVA)-induced allergic airway inflammation (AAI) (38) in AhR-deficient (AhR^-/-^) and wild-type mice. In addition, the effects of AhR activation during AIT on its therapeutic outcome are investigated. To agonistically engage the AhR during AIT, the synthetic ligand 10-chloro-7H-benzimidazo[2,1-a]benzo[de]iso-quinolin-7-one (10-Cl-BBQ) was chosen (39). It is a non-toxic ligand that can induce strong AhR activation at a nanomolar concentration while being rapidly metabolized (40). 10-Cl-BBQ was shown to induce Tregs and suppress IL17A production in Th17 cells (39, 40), making it an attractive candidate as an adjuvant in AIT.

## 2 Results

### 2.1 The AhR is not essential for the success of AIT in murine AAI

**Figure 1:**
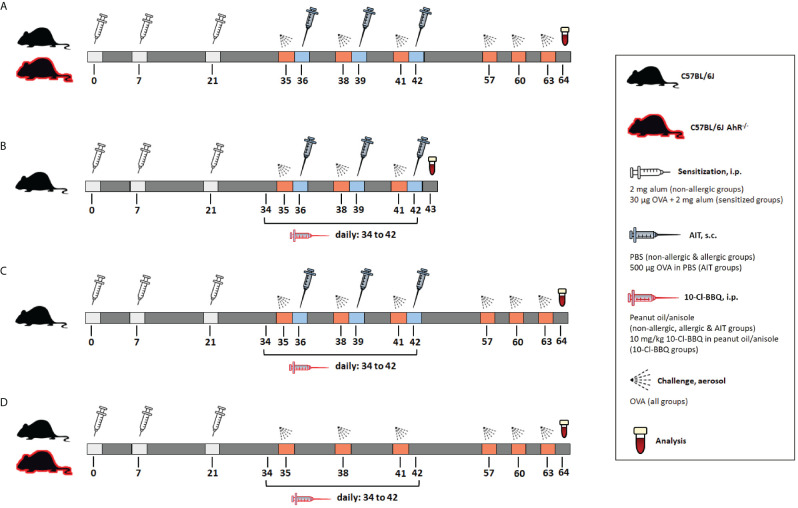
Schematic overview of the murine models used in the study. **(A)** Model of AIT in OVA-induced AAI, performed in AhR^-/-^ and wild-type mice. **(B)** Model of murine AIT in OVA-induced AAI to investigate the short-term effects of 10-Cl-BBQ administration. **(C)** Model of murine AIT in OVA-induced AAI to explore the long-term impact of 10-Cl-BBQ administration. **(D)** Experimental design of 10-Cl-BBQ treatment in a murine model of OVA-induced AAI, performed in AhR^-/-^ and wild-type mice. AAI, allergic airway inflammation; AhR^-/-^, aryl hydrocarbon receptor knockout; AIT, allergen-specific immunotherapy; i.p., intraperitoneal; OVA, ovalbumin; s.c., subcutaneous.

**Figure 2:**
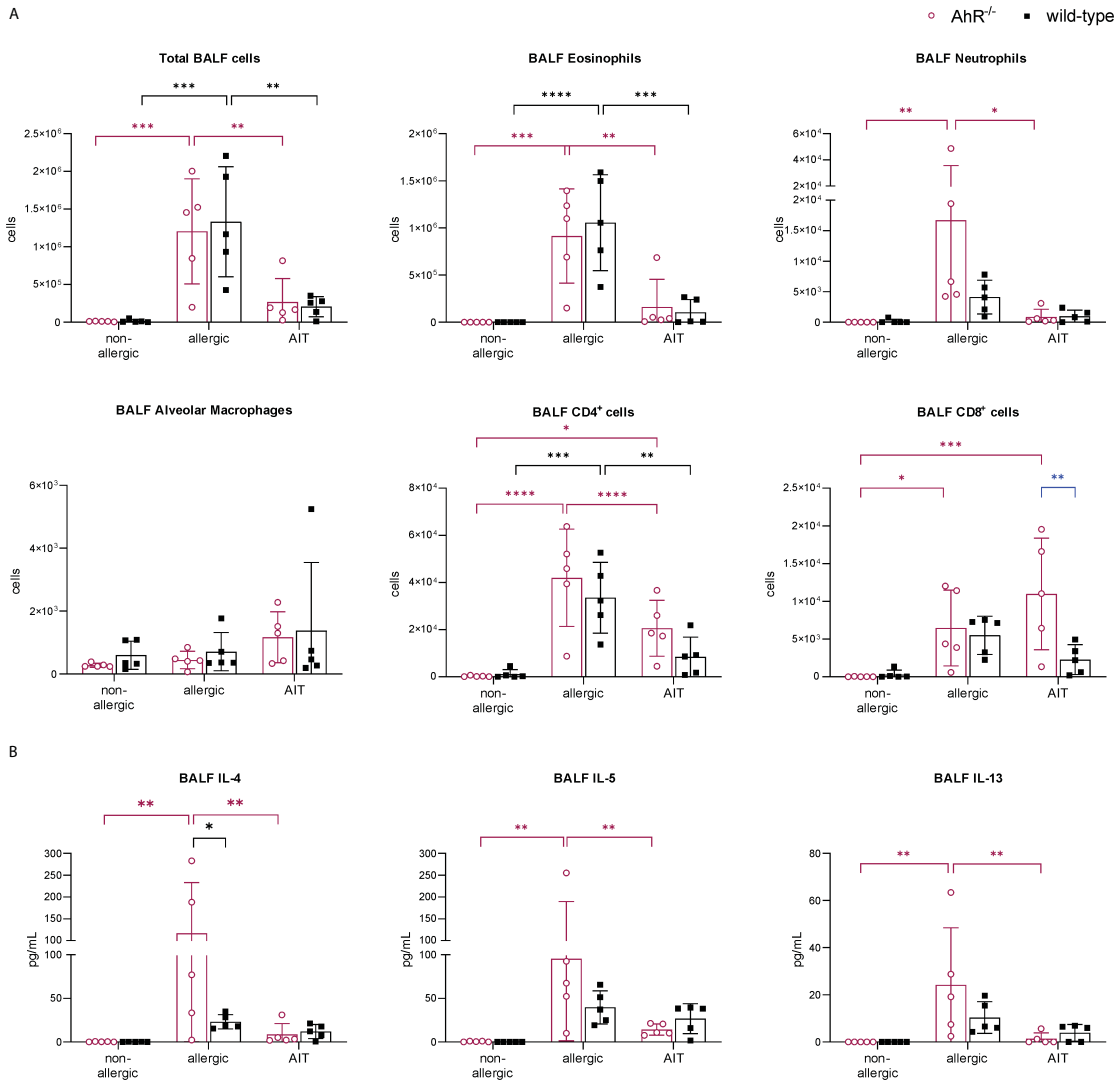
Effects of experimental AIT on BALF cell counts and cytokine levels in wild-type vs. AhR^-/-^ mice. **(A)** Total and differential BALF cell counts and **(B)** cytokine levels in the BALF measured at the end (day 64) of the experiment (n = 5). Data analyzed with 2-way ANOVA with Tukey’s multiple comparisons test or Sidak’s multiple comparisons test, respectively. The bar charts show the mean with standard deviation. p-values of ≤.05, ≤.01, ≤.001, and ≤.0001 are shown as *, **, ***, and ****, respectively. AhR^-/-^, aryl hydrocarbon receptor knockout; AIT, allergen-specific immunotherapy; BALF, bronchoalveolar lavage fluid; CD, cluster of differentiation; IL, interleukin.

The importance of the AhR for successful AIT was investigated in a well-established murine AIT model of OVA-induced AAI (38) in AhR^-/-^ mice compared to wild-type mice (**Figure 1A**). As expected, total BALF cell counts and counts of eosinophils, neutrophils, CD4^+^ cells, and CD8^+^ cells were increased in the allergic groups of AhR^-/-^ and wild-type mice compared to the respective non-allergic control groups and cell counts did not differ significantly between both genotypes within a treatment group (**Figure 2A**). In both genotypes, AIT significantly reduced total BALF cells and the differential counts of eosinophils and CD4^+^ cells in a comparable manner. Furthermore, neutrophils were comparably reduced by AIT in both genotypes, however, the reduction was only significant in AhR^-/-^ mice. For CD8^+^ cells there was a significantly higher level in AIT-treated AhR^-/-^ compared to wild-type mice. Alveolar macrophages, in comparison, did not show any trend. (**Figure 2A**). While the levels of the Th2 cytokines IL-4, IL-5, and IL-13 were significantly increased in allergic AhR^-/-^ mice compared to non-allergic mice, this effect was less prominent and not significant in wild-type mice (**Figure 2B**). Despite these differences in the allergic groups, AIT treatment reduced the cytokines to a comparable level without significant differences between both genotypes.

**Figure 3:**
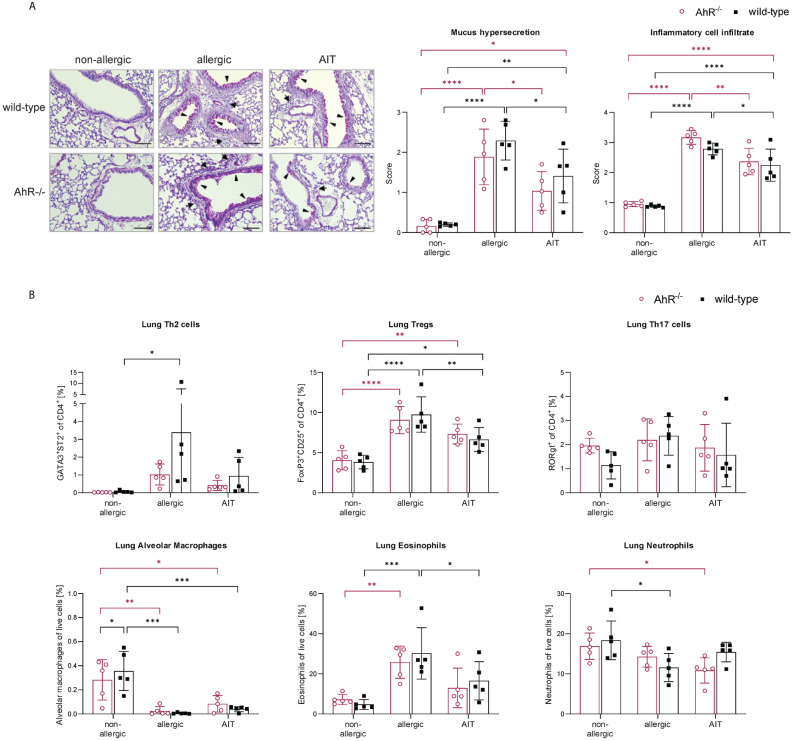
Effects of experimental AIT on lung histopathology and immune cells in wild-type vs. AhR^-/-^ mice. **(A)** Representative PAS staining of lung tissue (arrowheads: mucus hypersecretion; arrows: inflammatory cell infiltrate; scale bar: 100 µm) and scoring of mucus hypersecretion (n = 5) and inflammatory cell infiltrate (n = 5). **(B)** Flow cytometric analysis of T helper cell subsets, alveolar macrophages, eosinophils, and neutrophils isolated from lung tissue (n = 5). Data analyzed with 2-way ANOVA with Tukey’s multiple comparisons test or Sidak’s multiple comparisons test, respectively. The bar charts show the mean with standard deviation. p-values of ≤.05, ≤.01, ≤.001, and ≤.0001 are shown as *, **, ***, and ****, respectively. AhR^-/-^, aryl hydrocarbon receptor knockout; AIT, allergen-specific immunotherapy; PAS, periodic acid-Schiff; Th & Treg, T helper & regulatory cells.

**Figure 4:**
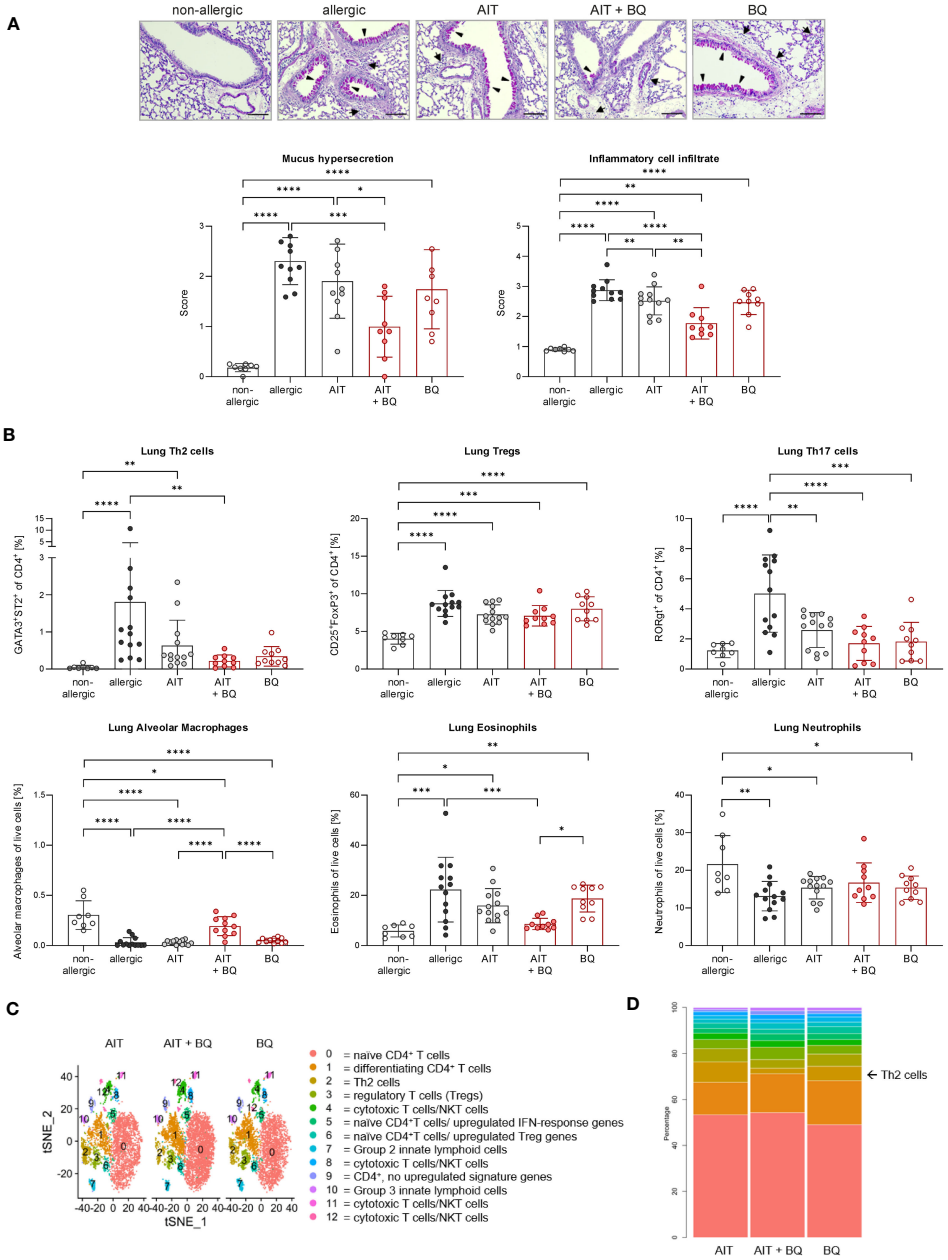
Long-term effects of the combination of AIT and 10-Cl-BBQ treatment on lung histopathology and immune cells. **(A)** Representative PAS staining of lung sections (arrowheads: mucus hypersecretion; arrows: inflammatory cell infiltrate; scale bar: 100 µm) and scoring of mucus hypersecretion (n = 10 – 13) and inflammatory cell infiltrate (n = 10 – 13). **(B)** Flow cytometric analysis of Th cell subsets, alveolar macrophages, eosinophils, and neutrophils isolated from lung tissue (n = 10 – 13). Gaussian and non-Gaussian distributed results were analyzed by 1-way ANOVA with Tukey’s test or Kruskal-Wallis test with Dunn’s test, respectively. The bar charts show the mean with standard deviation. p-values of ≤.05, ≤.01, ≤.001, and ≤.0001 are shown as *, **, ***, and ****, respectively. **(C)** Clusters in tSNE embedding after scRNA-seq analysis of enriched pulmonary CD4^+^ T cells and **(D)** percentages of clusters. AIT, allergen-specific immunotherapy; BQ, 10-Cl-BBQ; CD, cluster of differentiation; NKT cells, natural killer T cells; PAS, periodic acid-Schiff; scRNA-seq, single-cell RNA sequencing; Th & Treg, T helper & regulatory cells; tSNE, t-distributed stochastic neighbor.

Lung histological analysis demonstrated comparable efficacy of AIT in reducing mucus hypersecretion and inflammatory cell infiltration in mice of both genotypes compared to respective allergic groups (**Figure 3A**). While Tregs were significantly increased in the lung tissue of both allergic groups compared to the respective control groups, the increase of Th2 cells was only significant for allergic wild-type mice (**Figure 3B**). AIT slightly decreased both Th cell subsets, however, in a non-significant manner except for Tregs in wild-type mice, which showed a significant decrease. The levels of Th17 cells did not change significantly in any of the groups, although a trend to an increase of Th17 cells was observed in allergic wild-type mice. The percentages of lung alveolar macrophages were reduced in allergic and AIT-treated mice of both genotypes compared to the respective non-allergic control groups. Furthermore, eosinophils were significantly increased in allergic mice of both genotypes compared to non-allergic mice. AIT treatment reduced the level of eosinophils, although only significantly in wild-type mice. However, due to the high variation within this group the statistical significance (p=0,0443) should be considered with caution, rather underscoring the trend of eosinophil reduction. These observations are in line with results depicted in **Figure 4B**, where AIT alone also did not lead to a clear eosinophil reduction. The percentages of neutrophils changed only in a minor and rather unconcise manner (**Figure 3B**).

**Figure 5:**
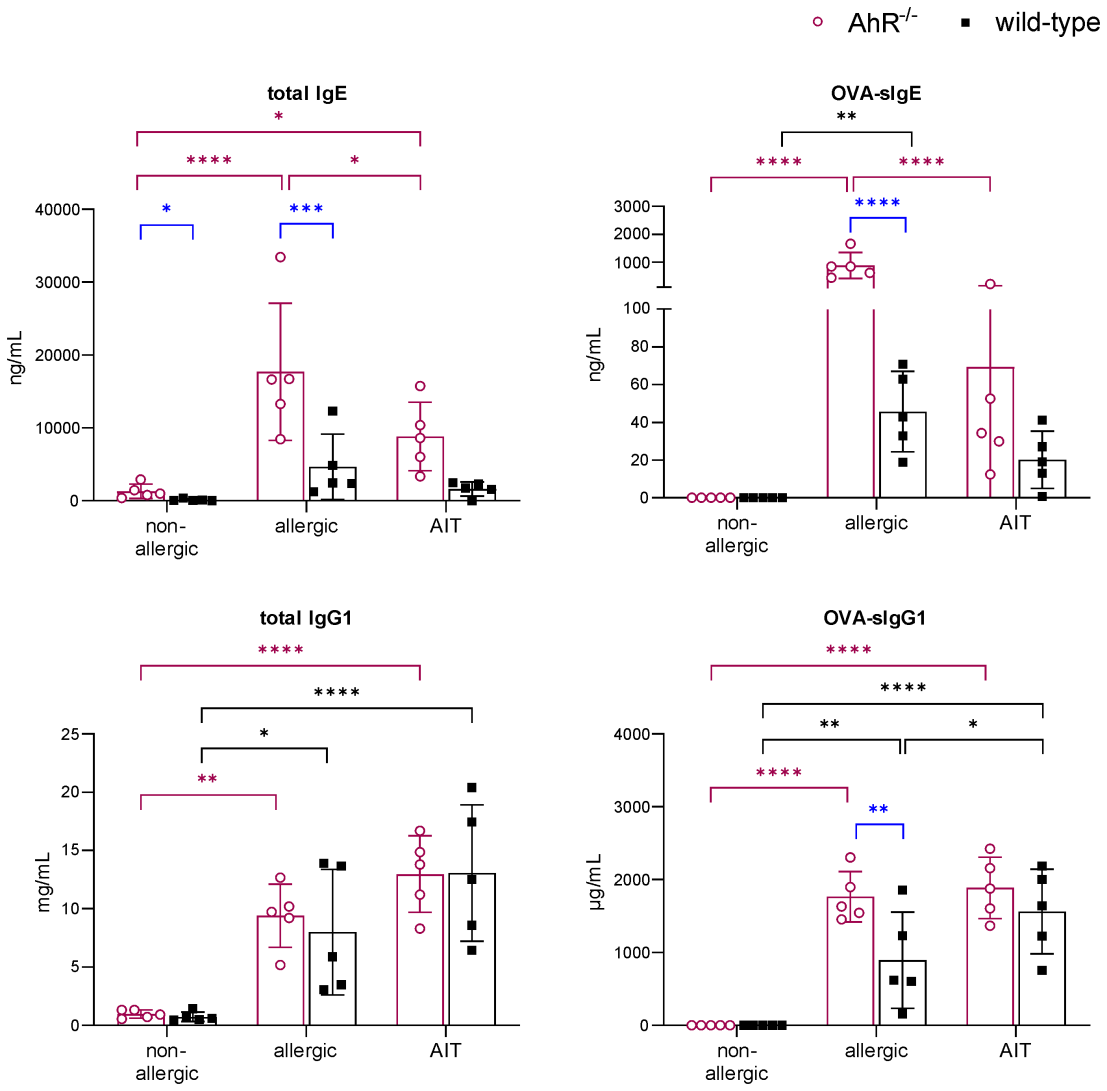
Effects of experimental AIT on immunoglobulin titers in wild-type vs. AhR^-/-^ mice. Analysis of total IgE, OVA-sIgE, total IgG1, and OVA-sIgG1 in serum samples collected at the end (day 64) of the experiment (n = 5). Data analyzed with 2-way ANOVA with Tukey’s multiple comparisons test or Sidak’s multiple comparisons test, respectively. The bar charts show the mean with standard deviation. p-values of ≤.05, ≤.01, ≤.001, and ≤.0001 are shown as *, **, ***, and ****, respectively. AhR^-/-^, aryl hydrocarbon receptor knockout; AIT, allergen-specific immunotherapy; Ig, immunoglobulin; OVA, ovalbumin; sIg, specific immunoglobulin.

Analysis of serum immunoglobulins revealed significantly higher levels of total and OVA-specific (OVA-s) IgE in allergic AhR^-/-^ compared to allergic wild-type mice (**Figure 5**). AIT led to a significant reduction of both parameters only in AhR^-/-^ mice, while wild-type mice showed a similar but non-significant trend. Total IgG1 levels did not differ between both genotypes within a treatment group. OVA-sIgG1 was significantly higher in allergic AhR^-/-^ and wild-type mice compared to the respective non-allergic groups. While AIT further increased OVA-sIgG1 in wild-type mice, it did not change in AhR^-/-^ mice after AIT.

Together, this data demonstrates that the ameliorating effect of experimental AIT on AAI is not dependent on the AhR.

### 2.2 The high-affinity AhR-agonist 10-Cl-BBQ improves the outcome of AIT in murine AAI

Given the role of the AhR in anti-inflammatory immune responses, we sought to investigate whether activation of the AhR specifically during AIT could represent an adjuvant strategy for AIT improvement, although involvement of the AhR *per se* is not a requirement for AIT success. Therefore, the impact of AhR activation on experimental AIT was addressed using the high-affinity agonist 10-Cl-BBQ. To evaluate the potentially tolerogenic effects of 10-Cl-BBQ-dependent AhR induction during AIT, treatment of allergic mice was performed with 10 mg/kg (39, 41), either in combination with AIT (AIT+BQ) or as a single-treatment strategy (BQ) (**Figures 1B, C**). Both treatment strategies were compared with the therapeutic outcome of standard AIT (AIT). To evaluate how long the effect of 10-Cl-BBQ would last, mice were sacrificed at two time points, one 24 h after the last 10-Cl-BBQ treatment (**Figure 1B**, day 43) and the other three weeks later following further challenges (**Figure 1C**, day 64).

**Figure 6:**
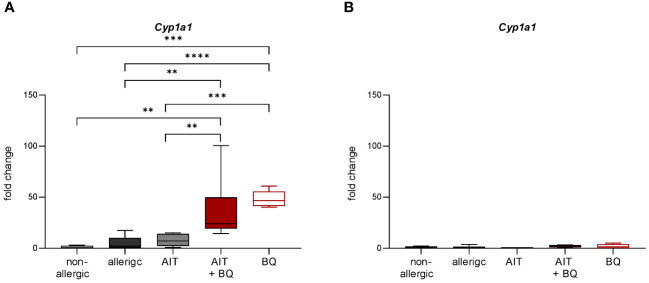
Effect of experimental AIT and 10-Cl-BBQ treatment on the expression of the AhR target gene *Cyp1a1* in murine lung tissue. **(A)** Gene expression analysis of *Cyp1a1* in lung tissue 24 h after the last 10-Cl-BBQ administration (day 43; **Figure 1B**); (n = 5 – 8). **(B)** Gene expression analysis of *Cyp1a1* in lung tissue 22 days after the last 10-Cl-BBQ administration and an additional OVA challenge phase (day 44; **Figure 1C**); (n = 10 – 13). Data analyzed by 1-way ANOVA with Tukey’s test. Data is displayed as box plots with whiskers indicating 1-99 percentile. p-values of ≤.01, ≤.001, and ≤.0001 are shown as **, ***, and ****, respectively. AIT, allergen-specific immunotherapy; BQ, 10-Cl-BBQ; *Cyp1a1*, Cytochrome P450 Family 1 Subfamily A Member 1.

On day 43, *Cyp1a1* expression was significantly induced in both 10-Cl-BBQ-treated groups compared to all other groups, which did not receive 10-Cl-BBQ, indicating 10-Cl-BBQ-dependent activation of the AhR (**Figure 6A**).

**Figure 7:**
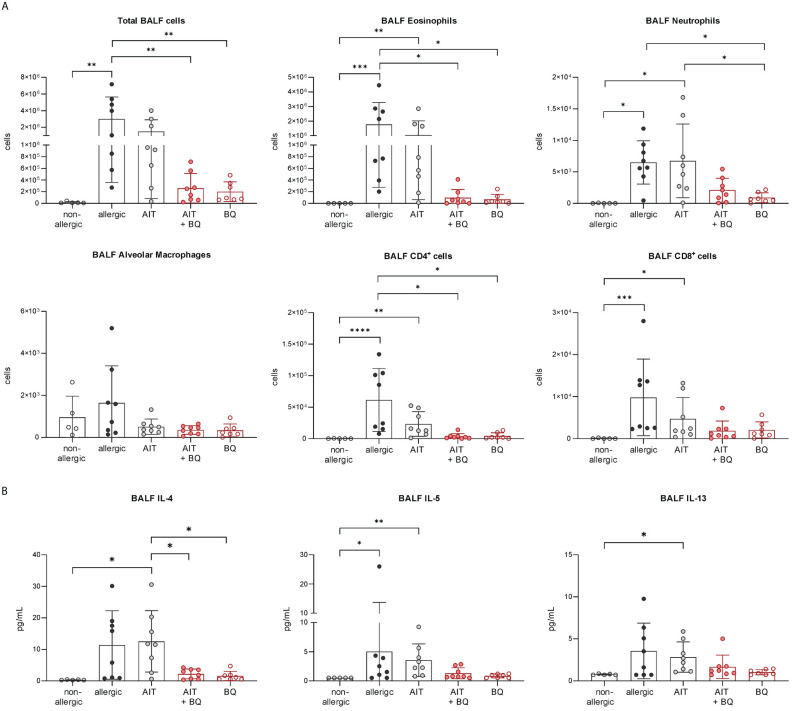
Short-term effects of 10-Cl-BBQ treatment and AIT on BALF cell counts and cytokine levels. **(A)** Total and differential BALF cell counts and **(B)** cytokine levels in the BALF measured at the end (day 43) of the experiment (n = 5 – 8). Gaussian and non-Gaussian distributed results were analyzed by 1-way ANOVA with Tukey’s test or Kruskal-Wallis test with Dunn’s test, respectively. The bar charts show the mean with standard deviation. p-values of ≤.05, ≤.01, ≤.001, and ≤.0001 are shown as *, **, ***, and ****, respectively. AIT, allergen-specific immunotherapy; BQ, 10-Cl-BBQ; BALF, bronchoalveolar lavage fluid; CD, cluster of differentiation; IL, interleukin.

**Figure 8:**
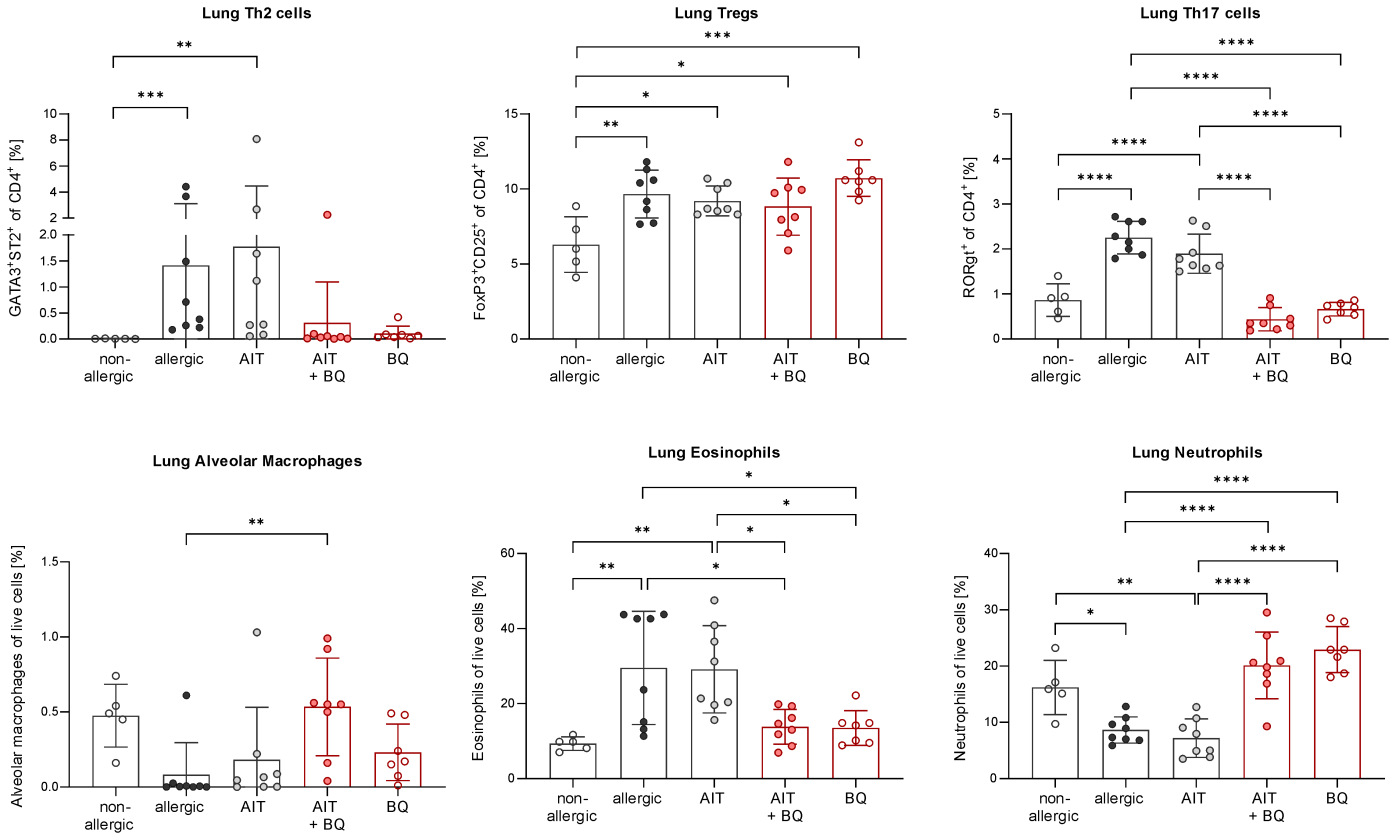
Short-term effects of 10-Cl-BBQ treatment and AIT on immune cells in the lung. Flow cytometric analysis of T helper cell subsets, alveolar macrophages, eosinophils, and neutrophils isolated from lung tissue (n = 5 – 8). Gaussian and non-Gaussian distributed results were analyzed by 1-way ANOVA with Tukey’s test or Kruskal-Wallis test with Dunn’s test, respectively. The bar charts show the mean with standard deviation. p-values of ≤.05, ≤.01, ≤.001, and ≤.0001 are shown as *, **, ***, and ****, respectively. AIT, allergen-specific immunotherapy; BQ, 10-Cl-BBQ.

Furthermore, total BALF cell counts and differential counts of eosinophils, neutrophils, CD4^+^ cells, and CD8^+^ cells were significantly increased in allergic mice compared to the non-allergic control group (**Figure 7A**). Notably, all these parameters (except CD8^+^ cells) were significantly reduced in the 10-Cl-BBQ-treated groups (AIT+BQ and BQ) compared to allergic mice, whereas effects of AIT were not detected at this time point. Alveolar macrophages did not show any clear trend (**Figure 7A**). A similar observation was found for the Th2 cytokine IL-4, while IL-5 and IL-13 were non-significantly reduced in the 10-Cl-BBQ-treated groups (**Figure 7B**). Moreover, compared to non-allergic mice, Th17 and Th2 cells were significantly increased in the lung tissue of allergic mice and those undergoing conventional AIT, while 10-CL-BBQ treatment led to a significant reduction of Th17 cells to a level observed in the non-allergic control mice. A similar but not significant trend was observed for Th2 cells (**Figure 8**). However, Tregs were increased by allergic sensitization but remained unchanged by AIT or 10-Cl-BBQ alone or the combined treatment (**Figure 8**). While the percentage of eosinophils in the lung tissue was significantly reduced in 10-Cl-BBQ-treated groups, the percentage of neutrophils, on the other hand, was increased. Additionally, the combined treatment increased the percentage of alveolar macrophages to a comparable level found in non-allergic mice (**Figure 8**). This data demonstrates the ability of the high-affinity AhR agonist 10-Cl-BBQ to induce *Cyp1a1* expression and alleviate characteristics of AAI shortly after its administration period.

**Figure 9:**
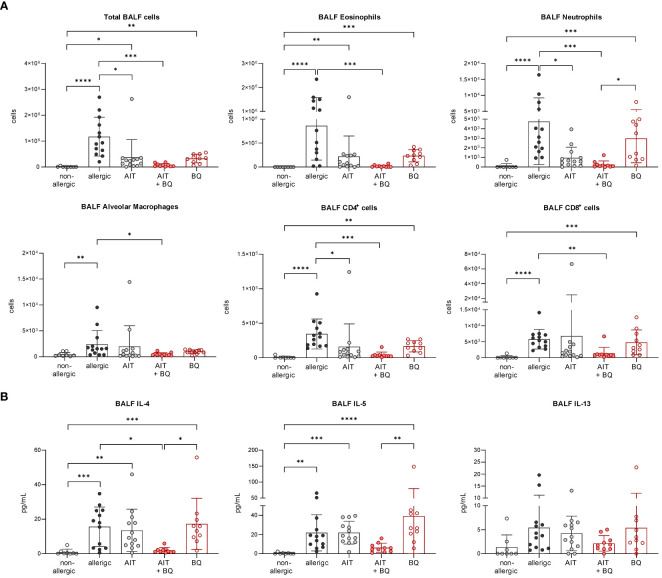
Long-term effects of the combination of AIT and 10-Cl-BBQ treatment on BALF cell counts and cytokine levels. **(A)** Total and differential BALF cell counts and **(B)** cytokine levels in the BALF measured at the end (day 64) of the experiment (n = 10 – 13). Gaussian and non-Gaussian distributed results were analyzed by 1-way ANOVA with Tukey’s test or Kruskal-Wallis test with Dunn’s test, respectively. The bar charts show the mean with standard deviation. p-values of ≤.05, ≤.01, ≤.001, and ≤.0001 are shown as *, **, ***, and ****, respectively. AIT, allergen-specific immunotherapy; BQ, 10-Cl-BBQ; CD, cluster of differentiation; IL, interleukin.

In contrast on day 64, 22 days after the last AIT and/or 10-Cl-BBQ injection, *Cyp1a1* induction could no longer be detected (**Figure 6B**), most likely because *Cyp1a1* is considered an immediate response gene after AhR activation (19, 42). Notably, both AIT alone and AIT combined with 10-Cl-BBQ administration significantly reduced total BALF cell counts. However, the effect was more prominent in the latter group (**Figure 9A**). Additionally, the reduction of the differential counts of eosinophils, neutrophils, alveolar macrophages, CD4^+^ cells, and CD8^+^ cells was more pronounced after the combined treatment compared to AIT alone (**Figure 9A**). The single administration of 10-Cl-BBQ did not lead to any significantly reduced cell counts in BALF, although the reduction of total BALF cell and differential eosinophil counts was strongly pronounced. Moreover, the combination of AIT and 10-Cl-BBQ administration significantly reduced IL-4 levels in the BALF compared to allergic mice while also showing a tendency to reduce IL-5 and IL-13. Interestingly, AIT or 10-Cl-BBQ single-treatment did not affect the levels of these Th2 cytokines (**Figure 9B**).

Lung histological analysis demonstrated a significant reduction of mucus hypersecretion and inflammatory cell infiltration for the treatment strategy combining AIT with 10-Cl-BBQ administration compared to allergic mice but for none of the single treatment strategies (**Figure 4A**). In addition, lung Th2 cells were significantly reduced solely by the combined treatment strategy, whereas single treatments demonstrated a clear but not statistically significant reduction. In contrast, Th17 cells were strongly reduced by all treatment regimens compared to the allergic control group, although no effect on Tregs was observed (**Figure 4B**). Interestingly, and in stark contrast to the single treatments, the combination of AIT with 10-Cl-BBQ administration restored the percentage of lung alveolar macrophages nearly to a non-allergic level. While the different treatment regimens did not influence lung neutrophils, eosinophils were significantly reduced only by the combined treatment compared to allergic mice (**Figure 4B**).

To further investigate whether AIT and/or 10-Cl-BBQ treatment elicits any changes in major Th cell populations or particular transcriptional profiles, scRNA-seq analysis of enriched CD4^+^ T cells isolated from lung tissue was performed. Cluster analysis with tSNE embedding was done for AIT-, 10-Cl-BBQ-, and AIT+10-Cl-BBQ-treated mice and clusters assigned to respective cell populations by analysis of the most differentially expressed genes. CD4^+^ T cells were enriched with a bead-based isolation kit. Hence, other cell populations, such as innate lymphoid, cytotoxic, and natural killer T cells, were also detected. In general, treatment with 10-Cl-BBQ did not induce other CD4^+^ T cell populations than observed after AIT (**Figure 4C**). Among all analyzed CD4^+^ T cell populations, no relevant differences in gene expression profiles between the different treatment strategies were observed (data not shown). The most striking finding, a reduced percentage of Th2 cells by the combined treatment with AIT and 10-Cl-BBQ compared to the single treatment strategies (**Figure 4D**), underlined the previous observations (**Figure 4B**).

**Figure 10:**
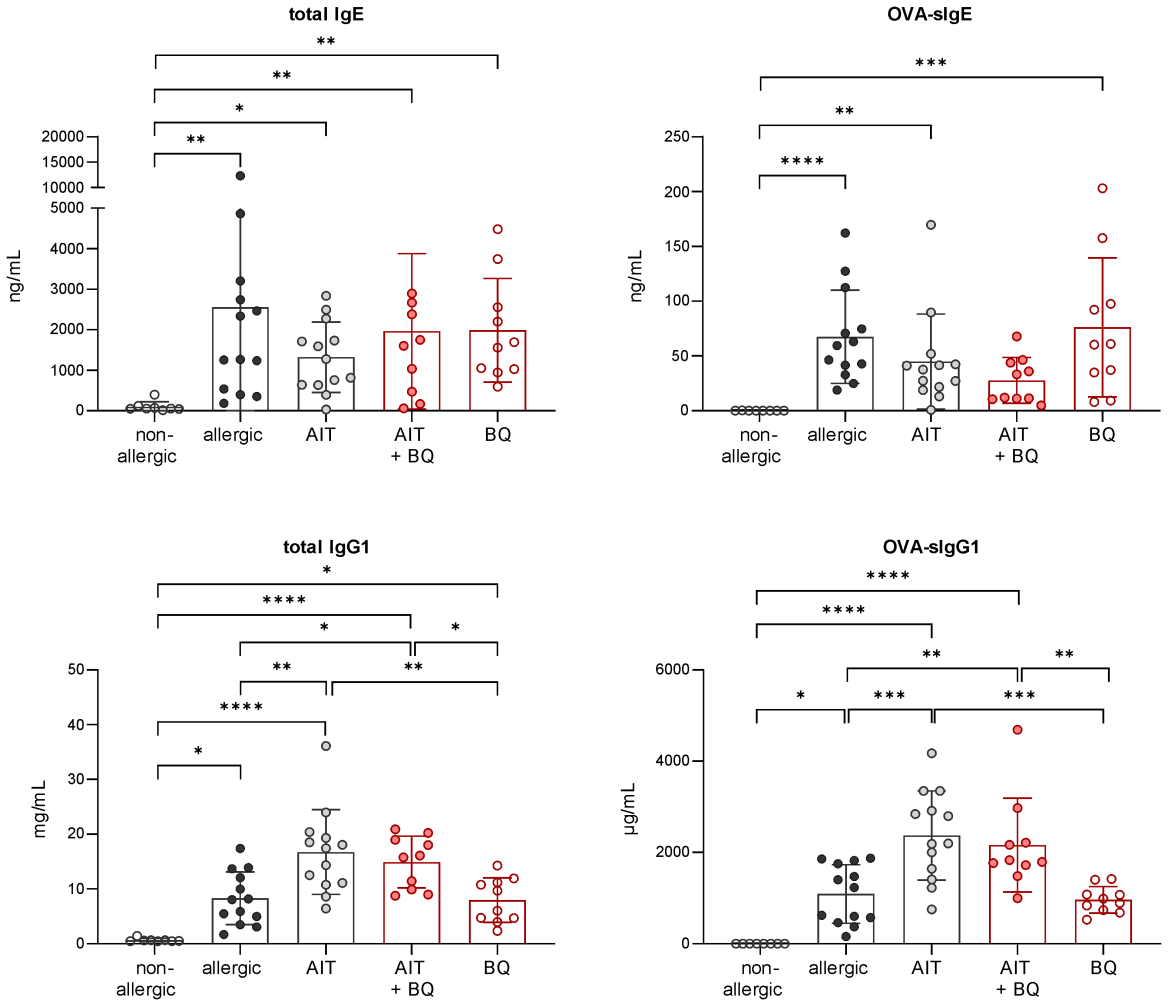
Long-term effects of the combination of AIT and 10-Cl-BBQ treatment on immunoglobulin titers. Analysis of total IgE, OVA-sIgE, total IgG1, and OVA-sIgG1 in serum samples collected at the end (day 64) of the experiment (n = 10 – 13). Gaussian and non-Gaussian distributed results were analyzed by 1-way ANOVA with Tukey’s test or Kruskal-Wallis test with Dunn’s test, respectively. The bar charts show the mean with standard deviation. p-values of ≤.05, ≤.01, ≤.001, and ≤.0001 are shown as *, **, ***, and ****, respectively. AIT, allergen-specific immunotherapy; BQ, 10-Cl-BBQ; Ig, immunoglobulin; OVA, ovalbumin; sIg, specific immunoglobulin.

Independent of the treatment strategy, the total IgE levels did not differ significantly from the allergic group. However, there was a tendency towards reduced OVA-sIgE after combined treatment. Total IgG1 and OVA-sIgG1 levels were higher in mice treated with AIT or the combination of AIT and 10-Cl-BBQ administration than in allergic mice and those receiving 10-Cl-BBQ as a single treatment (**Figure 10**).

These results demonstrate that combining AIT with 10-Cl-BBQ administration improves AIT outcome.

### 2.3 The AhR mediates the effects of 10-Cl-BBQ on AAI

**Figure 11:**
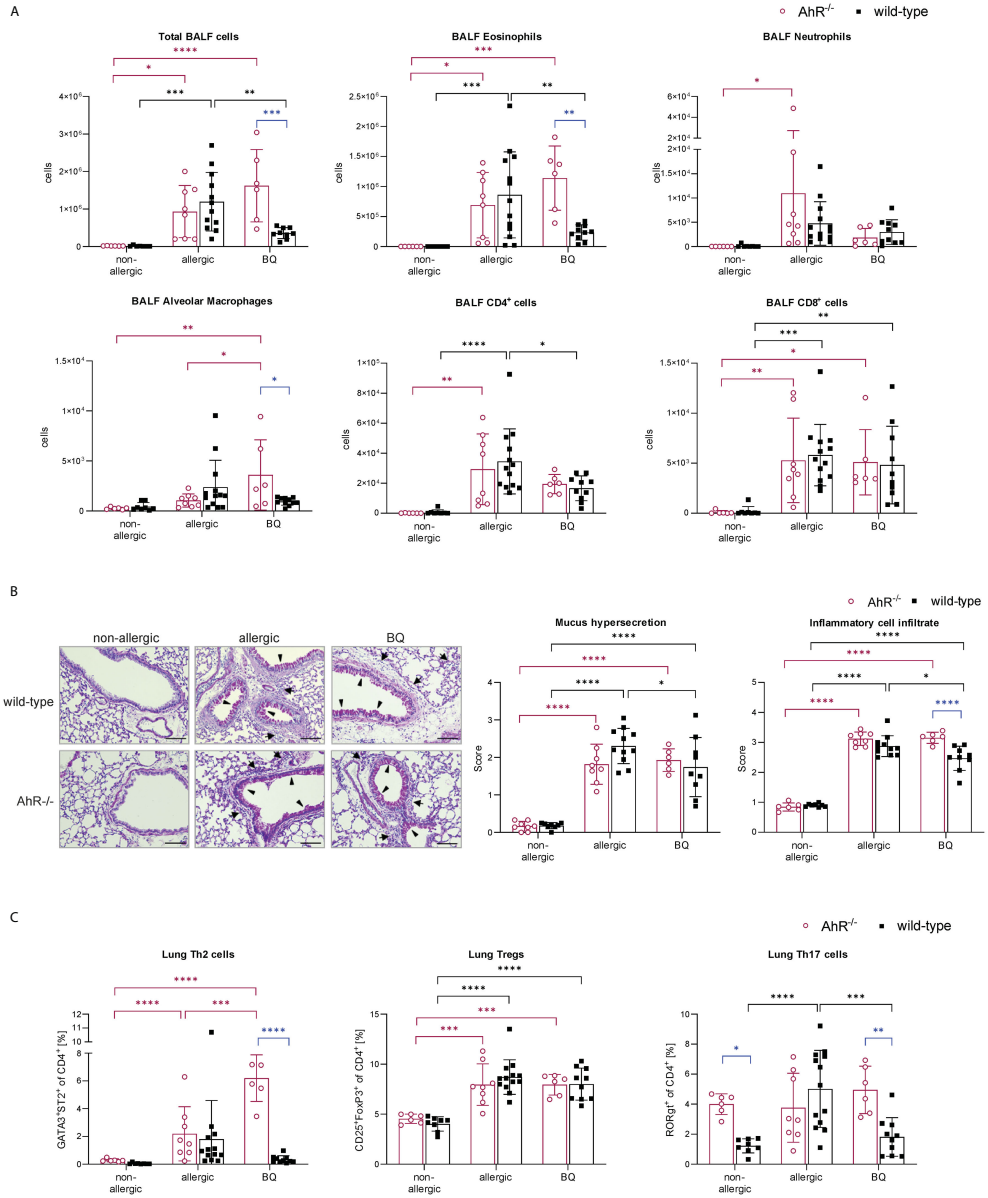
AhR-dependency of 10-Cl-BBQ administration on allergic airway inflammation. **(A)** Total and differential BALF cell counts at the end (day 64) of the experiment (n = 6 – 13). **(B)** Representative PAS staining of lung sections (arrowheads: mucus hypersecretion; arrows: inflammatory cell infiltrate; scale bar: 100 µm) and scoring of mucus hypersecretion (n = 6 – 13) and inflammatory cell infiltrate (n = 6 – 13). **(C)** Flow cytometric analysis of Th cell subsets from lung tissue (n = 6 – 13). Data analyzed by 2-way ANOVA with Tukey’s multiple comparisons test or Sidak’s multiple comparisons test, respectively. The bar charts show the mean with standard deviation. p-values of ≤.05, ≤.01, ≤.001, and ≤.0001 are shown as *, **, ***, and ****, respectively. AhR, aryl hydrocarbon receptor; BALF, bronchoalveolar lavage fluid; BQ, 10-Cl-BBQ; CD, cluster of differentiation; OVA, ovalbumin; PAS, periodic acid-Schiff; Th & Treg, T helper & regulatory cells.

To further verify that the AhR is not only activated by 10-Cl-BBQ (**Figure 6A**) but also responsible for the observed immunomodulatory effects, OVA-allergic AhR^-/-^ and wild-type mice were treated with 10-Cl-BBQ and compared with untreated non-allergic and allergic mice (**Figure 1D**). Notably, total BALF and differential eosinophil cell counts were significantly reduced in 10-Cl-BBQ-treated wild-type mice compared to allergic mice, which was not observed in AhR^-/-^ mice (**Figure 11A**). In contrast to eosinophils, which were significantly increased in allergic mice of both genotypes compared to non-allergic mice, neutrophils were only significantly higher in allergic AhR^-/-^. However, 10-Cl-BBQ treatment did not significantly influence the neutrophil cell population. For alveolar macrophages, there is a trend to increased cell counts upon allergic sensitization (allergic & 10-Cl-BBQ groups) in both genotypes. 10-Cl-BBQ treatment in AhR^-/-^ mice further increases this level, however, there is a higher variability in cell counts. CD4^+^ and CD8^+^ cells were significantly increased in allergic mice of both genotypes. Although not statistically significant, the levels of CD4^+^ cells were also clearly elevated in 10-Cl-BBQ treated groups in comparison to the non-allergic mice. The administration of 10-Cl-BBQ exclusively reduced the level of CD4^+^ in wild-type mice, while in AhR^-/-^ mice, there was no clear trend due to high variation in the respective allergic group (**Figure 11A**).

Lung histological analysis demonstrated a comparable increase in mucus hypersecretion and inflammatory cell infiltration in allergic mice of both genotypes compared to the non-allergic control groups. However, both parameters were significantly reduced by 10-Cl-BBQ treatment exclusively in wild-type mice (**Figure 11B**). Strikingly, pulmonary Th2 cells, which were significantly increased in allergic AhR^-/-^ mice compared to the non-allergic control group, were even further increased after 10-Cl-BBQ treatment (**Figure 11C**). The levels of Th2 cells did not differ significantly in the different groups of wild-type mice. However, there was a tendency to induction in allergic mice and subsequent decline after 10-Cl-BBQ administration. The percentage of lung Th17 cells *per se* was higher in non-allergic AhR^-/-^ compared to wild-type mice and remained unchanged independent of the treatment group. However, in wild-type mice, the percentage increased significantly, which was reverted by 10-Cl-BBQ administration. In contrast, Tregs, which were significantly increased in the allergic groups of both genotypes, remained unchanged by the 10-Cl-BBQ treatment (**Figure 11C**).

In line with the results shown in **Figure 5**, allergic AhR^-/-^ mice showed increased immunoglobulin levels compared to wild-type mice. However, while OVA-sIgE and total IgG1 levels remained unchanged by 10-Cl-BBQ administration in wild-type mice, they were higher in 10-Cl-BBQ-treated AhR^-/-^ mice compared to allergic mice of the same genotype. In contrast, 10-Cl-BBQ administration did not influence the total IgE and OVA-sIgG1 levels in mice of both genotypes compared to the respective allergic groups (**Supplementary Figure 1**).

Altogether, these results demonstrate that treatment of allergic AhR^-/-^ mice with 10-Cl-BBQ does not ameliorate characteristics of AAI. Therefore, the beneficial effects of 10-Cl-BBQ observed in wild-type mice can be linked to activation of the AhR.

## 3 Discussion

This study shows for the first time that the AhR is not essential for the success of AIT in a murine model of OVA-induced AAI. The AhR is expressed in almost all tissues, and several studies have already described its effects on various immune cells (31, 33, 43). A link between activation of the AhR and anti-inflammatory properties has already been established in atopic dermatitis (43) and intestinal inflammation (44–46). Enhanced allergic phenotypes have been described for AhR^-/-^ mice in different models of AAI (47–49), which is in line with the observations of this study, where allergic AhR^-/-^ mice showed an increased grade of AAI with higher levels of Th2 cytokines, and total and OVA-sIgE, yet AIT reduced these markers to a ‘healthy’ wild-type level demonstrating that immune tolerance towards allergens during AIT is not affected by AhR-deficiency. Interestingly, and in line with other studies (49), the higher levels of BALF Th2 cytokines in allergic AhR^-/-^ mice do not necessarily correlate with a higher proportion of Th2 cells and, therefore, possibly originate from other cell populations, such as ILC2 (50), or from a differentially regulated immune response due to the lack of AhR signaling, leading to enhanced Th2-related responses (47).

The importance of AhR activation in DCs to promote Treg differentiation has been addressed extensively (51, 52), and it was shown that AhR-deficient DCs are not able to promote Treg differentiation *in vitro* and instead boost the generation of Th17 cells (33, 53). However, the AhR can modulate T cell differentiation and function indirectly via DCs and through direct transactivation and induction of epigenetic modifications (19). It has been shown that the AhR plays an important role in the development and function of natural FoxP3^+^ Tregs (nTregs), generated in the thymus, and in induced FoxP3^+^ Tregs (iTregs), generated in the periphery (54). The AhR impacts Tregs directly through the induction of FoxP3 (31) and by control of its epigenetic status, making it more accessible to the transcription machinery (26). Given the importance of the AhR for the induction of regulatory immune cell subsets and the principle of AIT, which is based on tolerance induction towards the allergy-eliciting allergens, it was somewhat unexpected that AhR deficiency did not change the therapeutic outcome of AIT in this study. However, future studies should address the Treg and DC phenotypes present in AhR^-/-^ mice undergoing AIT in more detail and investigate whether tolerogenic subsets differ in the absence of AhR signaling and which alternative signaling pathways participate in establishing allergen tolerance.

Although the presence of AhR was demonstrated to be dispensable for the success of AIT in experimental murine AAI, its diverse roles in anti-inflammatory immune responses make its targeting during AIT an attractive adjuvant strategy. Therefore, the impact of AhR engagement on the efficacy and immunomodulatory potential of AIT was addressed using the high-affinity AhR ligand 10-Cl-BBQ, which was demonstrated to induce Tregs and suppress IL17A production in Th17 cells (39, 40). Significantly induced *Cyp1a1* expression immediately after the administration period confirmed the 10-Cl-BBQ-dependent activation of the AhR. 10-Cl-BBQ exhibited a reducing effect on BALF cells and cytokines. Interestingly, the 10-Cl-BBQ treatment decreased lung Th17 cells, eosinophils, and, to a lesser extent, Th2 cells, while neutrophils and alveolar macrophages were restored to the level of non-allergic mice. These results indicate notable immediate effects of 10-Cl-BBQ on relevant parameters of AAI and emphasize the anti-inflammatory and immunomodulatory potential of AhR activation. In line with previous findings, which showed that modulating effects of AIT on cellular responses in the employed OVA model are not detectable immediately after the treatment period (38), at this time point of analysis, no apparent differences between allergic mice and those treated by AIT alone could be recognized.

Interestingly, 10-Cl-BBQ was not only able to reduce parameters of AAI short-term after its administration but also improved the efficacy of AIT at a later time point. The therapeutic strategy combining AIT with administering 10-Cl-BBQ further reduced AAI, including parameters such as total and differential cell counts and cytokine levels in the BALF, mucus hypersecretion, and inflammatory cell infiltration into the lung tissue. Of note, single treatment with 10-Cl-BBQ at this time point (day 64) clearly affected the level of eosinophils in the BALF and of Th2 cells in lung tissue, although not significantly in all experiments, while other cell populations were less impacted. In contrast, immediate effects of 10-Cl-BBQ directly after the administration period (day 43) were detected in most of the investigated cell populations, indicating that its acute anti-inflammatory impact seems to be linked to ligand availability in the system. The serum half-life of 10-Cl-BBQ has been reported as approx. 2 h (39). Therefore, the long-term effects of a single treatment on eosinophils and Th2 cells are quite interesting and worth following up on. Interestingly, a role for AhR signaling in tissue adaption of intestinal eosinophils has already been described (55).

The combination of AIT and 10-Cl-BBQ treatment restored lung alveolar macrophages to a comparable percentage found in non-allergic mice. Given that neither AIT nor 10-Cl-BBQ administration alone showed a similar impact, leads to the assumption that 10-Cl-BBQ-dependant AhR activation during AIT can mediate the level of alveolar macrophages in the lung tissue. Alveolar macrophages are mostly of embryonic origin and maintain themselves autonomously through *in situ* self-renewal, while under inflammatory conditions, it was shown that they can be replenished by monocyte-derived cells (56). Interestingly, the AhR has been assigned an important role as a molecular switch for the fate of monocytes (32) and, in allergic asthma, alveolar macrophages were found to regulate pro- and anti-inflammatory responses in the airways and links were established between lung macrophages, airway remodeling, and eosinophilic inflammation (57). Given the impact of the combined treatment on both cell populations, this described link between eosinophils and lung macrophages could be a particularly attractive and exciting putative target for future studies of AIT to elucidate.

10-Cl-BBQ administration demonstrated a reducing effect on Th cell subsets in the lung tissue, particularly on Th17 and, less pronounced, on Th2 cells, whereas Tregs remained unchanged. In CD4^+^ T cells, the AhR was identified as a direct target of the ligand 2,3,7,8-tetrachlorodibenzo-p-dioxin (TCDD) for the suppression of a murine acute graft-versus-host response (58, 59). Like TCDD, 10-Cl-BBQ is a potent AhR ligand but is rapidly metabolized and not cytotoxic to proliferating T cells (39). It was reported to influence the expression of CD25 and induce an AhR-dependent Treg phenotype (39), whereas, in this study, no 10-Cl-BBQ-induced changes of either CD25- or FoxP3-positive T cell levels were observed. AhR activation by high-affinity ligands mediates immunosuppression and is often associated with an increase in Tregs (40). However, in a murine model of type-1 diabetes, 10-Cl-BBQ almost completely prevented insulitis independent of FoxP3^+^ Tregs (40). Additionally, in the here applied AIT model of murine AAI, Tregs are already significantly elevated by the allergic sensitization *per se*, and 10-Cl-BBQ may not be able to increase percentages even further. Moreover, scRNA-seq analysis of CD4^+^ T cells isolated from lungs of mice undergoing the different treatment regimens did not reveal any striking differences in their transcriptional landscape. Still, scRNA-seq data confirmed the reduction in Th2 cells after 10-Cl-BBQ treatment as observed by flow cytometry, but no further differentially regulated gene patterns were found. This result suggests that the observed beneficial effects of 10-Cl-BBQ combined with standard AIT are most likely not caused by direct modulation of Th cells but by indirect effects involving other cell types, such as dendritic cells and/or macrophages, given the important role of the AhR in these cell types (20).

As expected, treatment of allergic AhR^-/-^ mice with 10-Cl-BBQ alone did not show any clear effects on AAI, underscoring the necessity of AhR engagement by 10-Cl-BBQ for any therapeutic effect. Intriguingly and rather unexpectedly, a pronounced increase in Th2 cells was observed upon 10-Cl-BBQ administration in AhR^-/-^ mice. It is not exactly clear what leads to this Th2 polarization. Potentially, off-target effects could be the reason for this. The ligand 10-Cl-BBQ has mostly been analyzed in its ability to activate the AhR receptor with a high affinity with little focus on alternative targets. Furthermore, ligand availability might be a relevant factor. In wild-type mice 10-Cl-BBQ is bound by the AhR receptor with very high affinity. This could ‘snatch’ the ligand away from off-target receptors, which, in combination with the engagement of AhR, could mask any potential off-targets in wild-type mice. In AhR^-/-^ mice, these effects (free ligand and no AhR engagement) could allow off-target effects to unfold. This must be investigated in further studies to evaluate the suitability of 10-Cl-BBQ as a putative adjuvant agent. Nevertheless, we were able to show an adjuvant effect of AhR engagement in combination with AIT in this study, underscoring the importance of identifying and characterizing adjuvant agents to develop novel therapeutic strategies to tackle allergic inflammation. Indeed, this line of research is very active with several other adjuvants showing promising results (38, 60–62).

Another important aspect to consider is the potential immunological effects of 10-Cl-BBQ metabolites. The metabolism of benzimidazoisoquinolines such as 10-Cl-BBQ in the body involves several pathways common to complex organic molecules (63, 64). Details on the specific metabolic pathways for 10-Cl-BBQ are not available, but its rapid clearance suggests involvement of typical phase I and II processes, such as oxidation and conjugation, to increase water solubility (65–68). Since the specific, potentially bioactive, metabolites of 10-Cl-BBQ have not been identified, we cannot rule out the possibility that some of its observed effects might not be solely due to AhR activation but could also be driven by other metabolite-related signaling pathways.

The AhR has been and still is intensively investigated as a potential therapeutic target in several immunological disorders, including allergic diseases. This study shows that targeting the AhR during AIT can help dampen inflammation and improve tolerogenic vaccination. The observed improvement after combining AIT with 10-Cl-BBQ treatment was directly linked to AhR activity by firstly showing a significantly enhanced *Cyp1a1* expression shortly after 10-Cl-BBQ administration and secondly by the absence of atherapeutic effect of 10-Cl-BBQ in allergic AhR^-/-^ mice. This knowledge must be evaluated carefully to develop further treatment options or improve current AIT protocols since the AhR is involved in various processes and expressed in multiple tissues and cell types. Nevertheless, this study demonstrates for the first time that, although the involvement of the AhR is not crucial for the success of AIT, its targeting during AIT may represent an attractive strategy to improve tolerogenic vaccination further and develop advanced AIT strategies.

## 4 Conclusion

**Figure 12:**
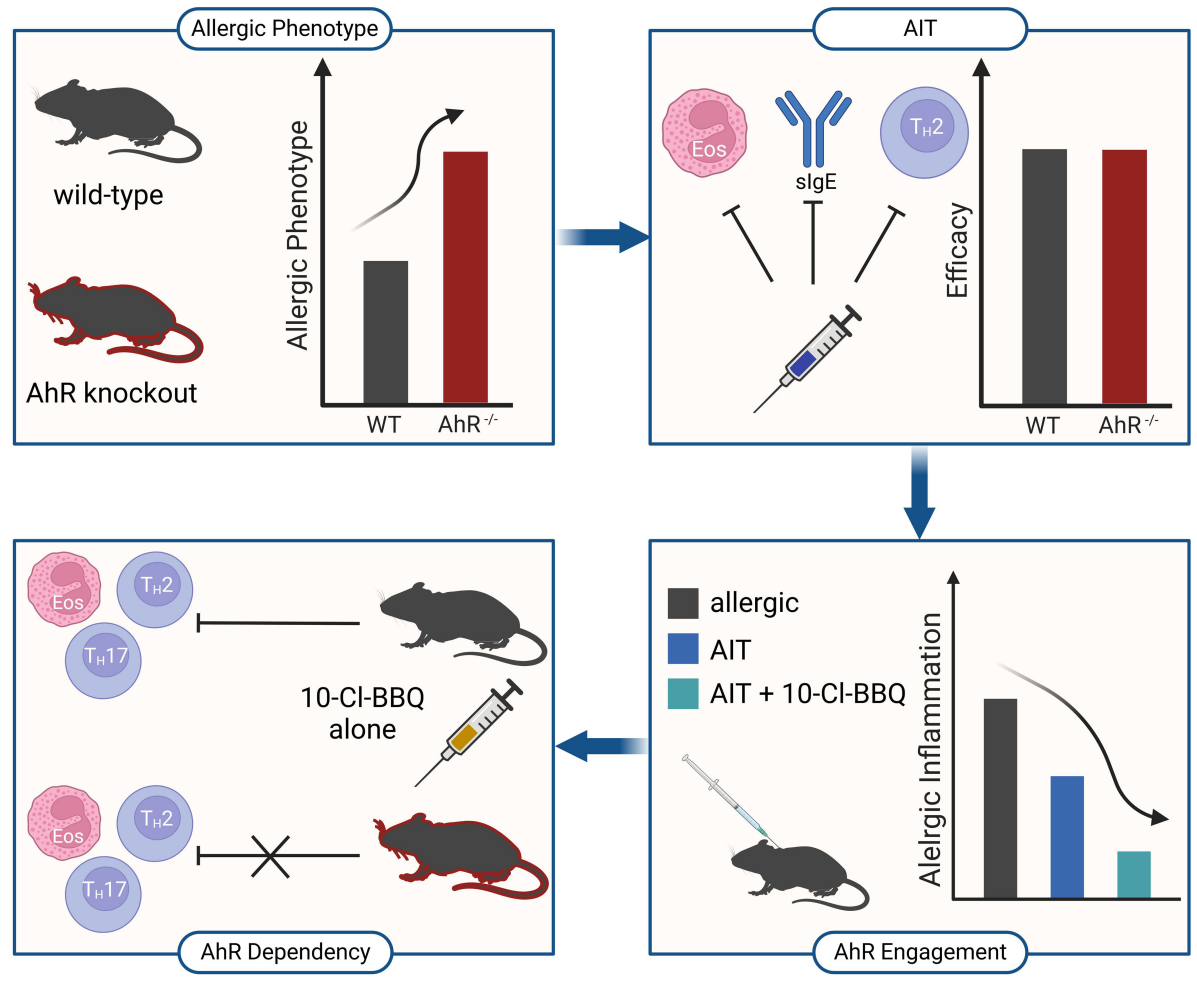
Schematic overview of the main findings of this study. AhR^-/-^, aryl hydrocarbon receptor knockout; AIT, allergen-specific immunotherapy; Eos, eosinophil; sIgE, specific immunoglobulin E; Th, T helper cell; WT, wild-type. Created with BioRender.com.

Although AhR knockout mice suffer from more robust allergic responses (**Figure 12**, top left), experimental AIT is comparably effective in wild-type and AhR-deficient mice (**Figure 12**, top right). Nevertheless, the high-affinity AhR agonist 10-Cl-BBQ promotes the efficacy of AIT in murine allergic airway inflammation (**Figure 12**, bottom right) in an AhR-dependent manner (**Figure 12**, bottom left). Therefore, AhR ligands might represent promising candidates as immunomodulators to improve the outcome of AIT, although successful AIT does not necessitate AhR engagement.

## 5 Materials and methods

### 5.1 Murine model of AIT in OVA-induced AAI

Female C57BL6/J and AhR-deficient (AhR^-/-^) (49, 69) mice aged 5-8 weeks (Charles River, Sulzfeld, Germany) were housed under specific pathogen-free conditions with food and water *ad libitum*. An overview of all murine models used in this study is provided in **Figure 1**. Allergic and AIT-treated mice were sensitized via intraperitoneal (i.p.) injections on days 1, 7, and 21 with 30 µg ovalbumin grade V (OVA) (Merck) and 2 mg aluminum hydroxide (alum) (Thermo Fisher Scientific) in 200 µl PBS. Non-allergic mice received injections of 2 mg alum in PBS (i.p.). After sensitization, the AIT groups were treated via subcutaneous (s.c.) injections of 500 µg OVA in 200 µl PBS on days 36, 39, and 42. The non-allergic and allergic groups, as well as groups treated with 10-Cl-BBQ only, received 200 µl PBS (s.c.). All mice were challenged with 1% nebulized OVA for 15 min on days 35, 28, 41, 57, 60, and 63 and euthanized on day 64. 10 mg/kg 10-Cl-BBQ (Bio-Techne GmbH, Wiesbaden, Germany), dissolved in anisole (Merck) and further diluted in peanut oil (Merck), combined with AIT or single-treatment strategy, was performed daily with i.p. injections from day 34 to 42. Mice of the non-allergic, allergic, and AIT groups received daily injections of the solvent (i.p.).

### 5.2 Isolation of BAL cells and pulmonary leukocytes

To isolate bronchoalveolar lavage (BAL) cells, the chest of euthanized mice was opened, the trachea cannulated, and the airways lavaged five times with 0.8 mL PBS. BAL cells were further analyzed via flow cytometry. To isolate pulmonary leukocytes, lungs were removed and chopped into small pieces. Digestion of lung tissue was performed in RPMI medium (Thermo Fisher Scientific) containing 1 mg/mL collagenase A and 100 µg/mL DNAse I (both from Sigma-Aldrich, Merck, Darmstadt, Germany) for 30 min at 37° C. Digested lung tissue was mashed trough a 70 µm cell strainer. Cells were pelleted (400 g, 4° C, 5 min), resuspended in 5 mL 40% percoll in RPMI (v/v) solution, underlayered with 5 mL 80% percoll solution, and centrifuged (1600 g, RT, 15 min). Lymphocytes were collected from the interphase and washed with PBS.

### 5.3 Enrichment of CD4^+^ T cells

CD4^+^ T cells were enriched from pulmonary lymphocytes with the EasySep™ Mouse CD4^+^ T cell Isolation Kit and the EasyEights™ EasySep™ Magnet (both from STEMCELL Technologies, Vancouver, Canada), according to the manufacturer’s instructions.

### 5.4 Flow cytometry

The BD LSRFortessa™ flow cytometer (BD Biosciences, Franklin Lakes, NJ, USA) was operated with the FACSDiva software (BD Biosciences), and subsequent data analysis was performed with FlowJo V.7.2.2 (Tree star, Ashland, OR, USA).

#### 5.4.1 Staining of BALF cells

Isolated BALF cells were stained with a fixable viability dye for 10 min at 4° C. Cells were washed twice with FACS buffer (1% FCS in PBS) and labeled with antibodies against surface markers for 20 min at 4° C. All antibodies used are listed in **Supplementary Table 1**. Stained cells were washed twice, resuspended in FACS buffer, and analyzed using an LSRFortessa™ flow cytometer. The gating strategy of a representative sample is shown in **Supplementary Figure 2**.

#### 5.4.2 Staining of alveolar macrophages

Pulmonary leukocytes were incubated with Mouse BD Fc Block™ (BD Biosciences) for 10 min. The supernatant was removed, and cells were labeled with antibodies against respective surface antigens for 20 min at 4° C (**Supplementary Table 2**). Next, cells were washed twice with FACS buffer and resuspended in FACS buffer. Before analysis with a BD LSRFortessa™ flow cytometer, cells were stained with 7-amino-actinomycin (7-AAD) (Thermo Fisher Scientific) to exclude dead cells in the subsequent analysis. The gating strategy of a representative sample is shown in **Supplementary Figure 3**.

#### 5.4.3 Staining of Th cell populations

Pulmonary lymphocytes were stained with a fixable viability dye (for 10 min at 4° C to exclude dead cells). Cells were washed twice with FACS buffer (1% FCS in PBS) and labeled with antibodies against surface markers for 20 min at 4° C. All antibodies used are listed in **Supplementary Table 3**. Cells were washed twice with FACS buffer and fixed with the eBioscience™ FoxP3/Transcription factor kit (Thermo Fisher Scientific) for 30 min. After washing twice with fixation/permeabilization kit buffer, intracellular staining was performed for one hour at 4° C. Cells were washed once with fixation/permeabilization kit buffer and FACS buffer, resuspended in FACS buffer, and analyzed. The gating strategy of a representative sample is shown in **Supplementary Figure 4**.

### 5.5 Measurement of cytokine levels

Cytokine levels in the BALF were measured using BioLegend’s LEGENDplex T Helper Cytokine Panel (BioLegend, San Diego, CA, USA) according to manufacturers’ instructions and analyzed by BD LSRFortessa™ flow cytometer (BD Biosciences).

### 5.6 Measurement of immunoglobulins

Total IgG1 (tIgG1) levels were analyzed using the LEGENDplex™ Mouse Immunoglobulin Isotyping Panel (BioLegend) according to the manufacturer’s instructions. To detect OVA-specific IgG1 (OVA-sIgG1), plates were coated with 1 µg/mL ovalbumin grade V (Merck). OVA-sIgG1 was detected with a biotinylated anti-mouse IgG1 antibody (BD Biosciences). Total IgE (tIgE) was quantified with the BD Mouse IgE ELISA set (BD Biosciences) following the manufacturer’s instructions. OVA-specific IgE (OVA-sIgE) was analyzed using the LEGEND MAX™ Mouse OVA Specific IgE ELISA Kit (BioLegend) according to the manufacturer’s instructions.

### 5.7 Histological analysis

Lungs were excised after bronchoalveolar lavage (BAL). The left lobe was fixed in 4% buffered formalin and embedded in paraffin. Sections of 4 µm thickness were stained with hematoxylin-eosin (H&E) and periodic acid Schiff (PAS). Mucus hypersecretion and inflammatory cell infiltration were graded in a blinded fashion on a scale from 0 to 4 (0=none, 1=mild, 2=moderate, 3=marked, 4=severe), reflecting the degree of the pathological alteration (70).

### 5.8 Gene expression analysis

RNA was extracted from lung tissue or isolated lung lymphocytes using the Quick-RNA Miniprep Plus Kit (Zymo Research, Tustin, CA, USA) according to the manufacturer’s instructions. cDNA synthesis was performed with the RevertAid First Strand cDNA Synthesis Kit (Thermo Fisher Scientific) according to the manufacturer’s instructions. Gene expression levels were determined with the LightCycler^®^ 480 SYBR Green I Master (Roche, Basel, Switzerland), according to the manufacturer’s instructions. The primers used for gene expression analysis are listed in **Supplementary Table 4**.

### 5.9 Statistical analysis

GraphPad Prism8 (GraphPad Software, La Jolla, CA, USA) was used for statistical analysis. Specifically, Gaussian distribution was tested by D’Agostino & Pearson omnibus normality test. Gaussian and non-Gaussian distributed results were further analyzed by 1-way ANOVA with Tukey’s test or Kruskal-Wallis test with Dunn’s test, respectively. Results of wildtype and AhR^-/-^ mice were analyzed by 2-way ANOVA with Tukey’s test or Sidak’s test, respectively. p-values of ≤0.05, ≤0.01, ≤0.001, and ≤0.0001 are shown as *, **, ***, and **** or +, ++, +++, and ++++.

### 5.10 Single-cell RNA sequencing

All libraries for scRNA-seq of enriched CD4^+^ T cells were prepared according to the manual (CG000315 Rev A) of the Chromium Next GEM Single Cell 3’ Reagent Kit v3.1 (Dual Index) provided by 10x Genomics B.V. (Leiden, The Netherlands). A cell recovery of 10.000 was targeted. Sequencing was performed in the Core Facility Genomics at Helmholtz Munich (Munich, Germany) using the NovaSeq 6000 system (Illumina, San Diego, CA, USA). Libraries were denatured and diluted according to Illumina sequencing platform recommendations and sequenced using the following run configuration: Read 1: 28 cycles, i7 index: 10 cycles, i5 index: 10 cycles, Read 2: 90 cycles.

Data were processed on the GalaxyEU web-based platform for reproducible computational analysis (71). GRCm39 (Genome Reference Consortium Mouse Build 39 from 2020/06/24 - mm39) was used as the reference genome, and mapping, demultiplexing, and gene quantification were performed using RNA STARSolo (72). The following settings were chosen: for UMI-deduplication, the CellRanger 2-4 algorithm was applied. Multimatching of cell barcodes to the whitelist was allowed for cell barcodes with N-bases. UMIs with lower-counts mapping to more than one gene were removed. Using an EmptyDrops algorithm (lower-bound threshold: 100, false discovery rate: 0.01), cell filtering was performed by DropletUtils (73).

The R package Seurat 4.1.0 was used for data analysis (74, 75). Droplets with fewer than 50 or more than 5500 feature RNAs were excluded. The percentage of mitochondrial RNA per droplet was not allowed to exceed 12%. Droplets with more than 55% ribosomal RNA were also excluded from the analyses. Before determining the 2000 most important variable features using the ‘vst’ selection method, the individual data sets were normalized and merged. Data were scaled, and principal component analysis was performed for dimensionality reduction. For t-SNE and UMAP embeddings, only statistically significant principal components were considered (JackStraw approach). K nearest neighbors (k = 20), nearest neighbor graphs, and shared nearest neighbors were computed. Using a shared nearest neighbor modularity optimization-based clustering algorithm (resolution = 0.5), clusters were identified. The 40 most differentially expressed genes per cluster were calculated and based on this, clusters were assigned to cell types (74). Differentially expressed genes were calculated using the Wilcoxon rank sum test and a log fold change threshold of 0.25.

## Data availability statement

The datasets presented in this study can be found in online repositories. The names of the repository/repositories and accession number(s) can be found below: E-MTAB-14030 (ArrayExpress).

## Ethics statement

The animal study was approved by the government of the district of Upper Bavaria, Germany (ethical approvals: 55.2-1-54-2532-50-2017, 55.2-2532.Vet_02-17-222, and 55.2-2532.Vet_02-20-098). The study was conducted in accordance with the local legislation and institutional requirements.

## Author contributions

SH: Formal Analysis, Investigation, Methodology, Visualization, Writing – original draft. FA: Formal Analysis, Investigation, Methodology, Visualization, Writing – review & editing. JG (3rd author): Investigation, Visualization, Writing – original draft. CG: Investigation, Methodology, Validation, Writing – review & editing. AH: Investigation, Writing – review & editing. BS: Investigation, Writing – review & editing. JG (7th author): Investigation, Writing – review & editing. JB: Conceptualization, Writing – review & editing. BOS: Visualization, Writing – review & editing. DK: Supervision, Writing – review & editing. FF: Supervision, Writing – review & editing. CO: Supervision, Writing – review & editing. CS-W: Conceptualization, Funding acquisition, Supervision, Writing – review & editing. SB: Conceptualization, Funding acquisition, Supervision, Visualization, Writing – original draft, Writing – review & editing.
