# Supplementary material for: Activation of the aryl hydrocarbon receptor improves allergen-specific immunotherapy of murine allergic airway inflammation: a novel adjuvant option?
Source: Front Immunol. 2024 Jun 10;15:1397072. doi: 10.3389/fimmu.2024.1397072 (PMC11194380; doi:10.3389/fimmu.2024.1397072)
Supplement: Supplementary file 1 [file DataSheet_1.docx]

Supplementary Material

Activation of the aryl hydrocarbon receptor improves allergen-specific immunotherapy of murine allergic airway inflammation: A novel adjuvant option?

**Sonja Heine, Francesca Alessandrini, Johannes Grosch, Carina Graß, Alexander Heldner, Benjamin Schnautz, Johanna Grosch, Jeroen Buters, Benjamin O. Slusarenko, Daniel Krappmann, Francesca Fallarino, Caspar Ohnmacht, Carsten B. Schmidt-Weber, Simon Blank***

*** Correspondence:** Simon Blank: simon.blank@tum.de

# Supplementary Figures and Tables

## Supplementary Figures


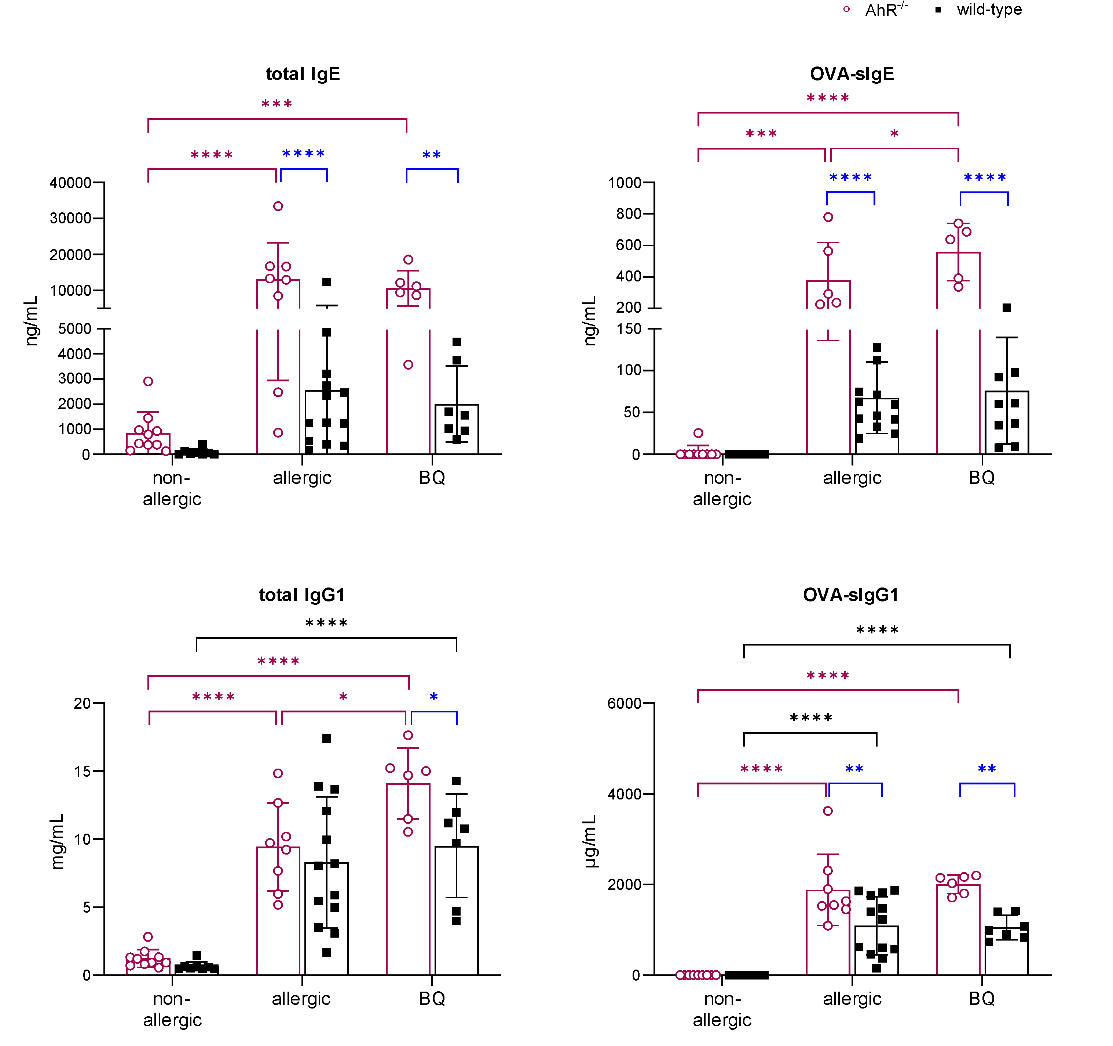


**Supplementary Figure 1.** AhR-dependency of 10-Cl-BBQ administration on immunoglobulin titers. Analysis of total IgE, OVA-sIgE, total IgG1, and OVA-sIgG1 in serum samples collected at the end (day 64) of the experiment (n = 6 – 13). Gaussian and non-Gaussian distributed results were analyzed by 1-way ANOVA with Tukey’s test or Kruskal-Wallis test with Dunn’s test, respectively. The bar charts show the mean with standard deviation. p-values of ≤.05, ≤.01, ≤.001, and ≤.0001 are shown as *, **, ***, and ****, respectively. BQ, 10-Cl-BBQ; Ig, immunoglobulin; OVA, ovalbumin; sIg, specific immunoglobulin.


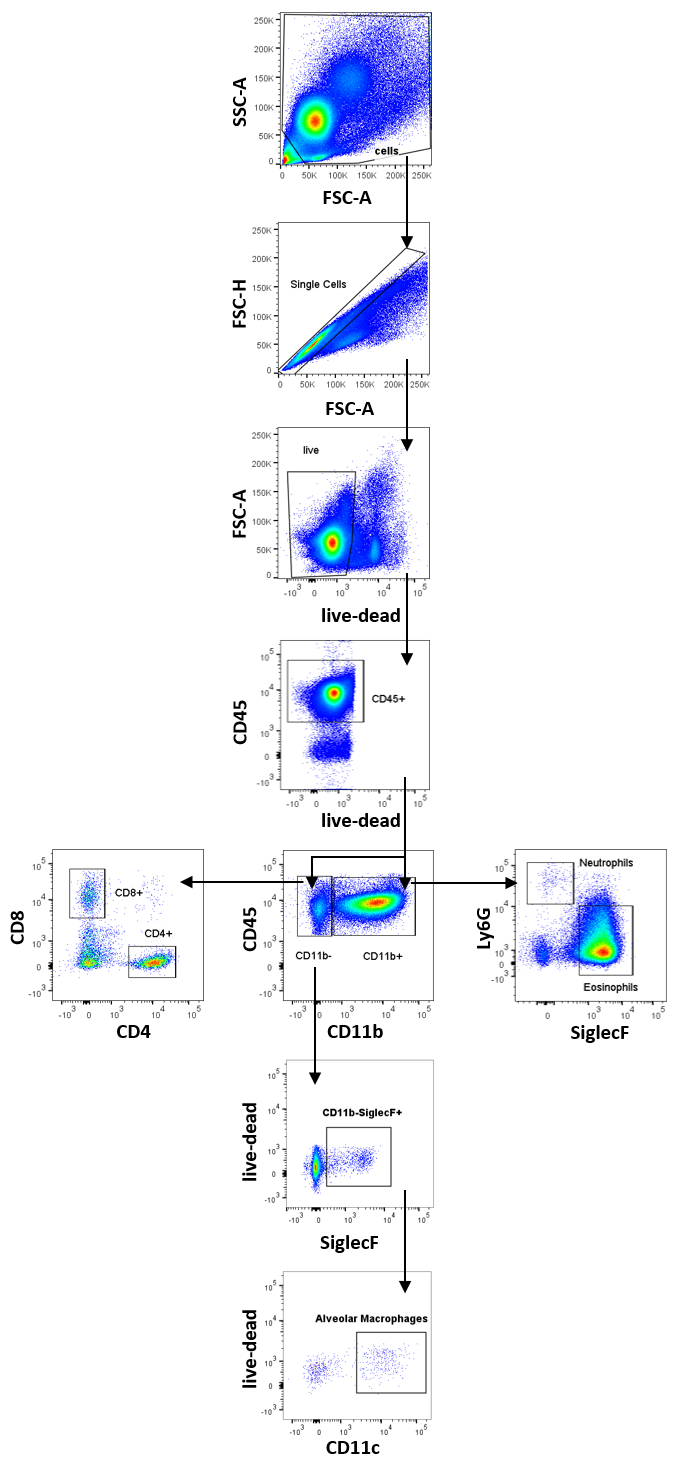


**Supplementary Figure 2.** Gating strategy for the characterization of cell populations in the BALF. Shown is the gating of one representative sample. BALF, bronchoalveolar lavage fluid.


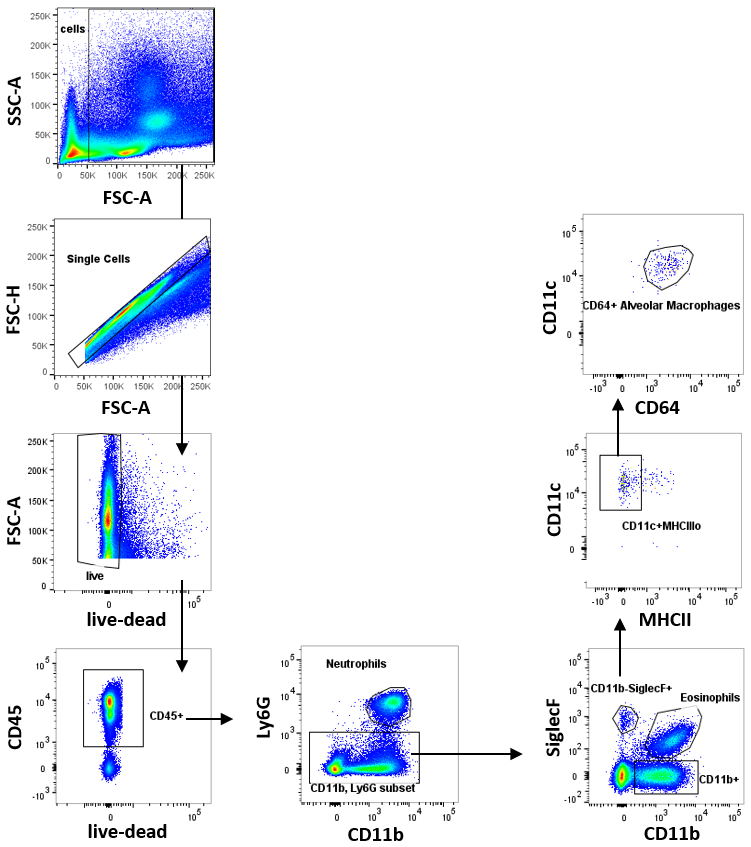


**Supplementary Figure 3.** Gating strategy for the characterization of alveolar macrophages in a pulmonary leucocyte population. Shown is the gating of one representative sample.


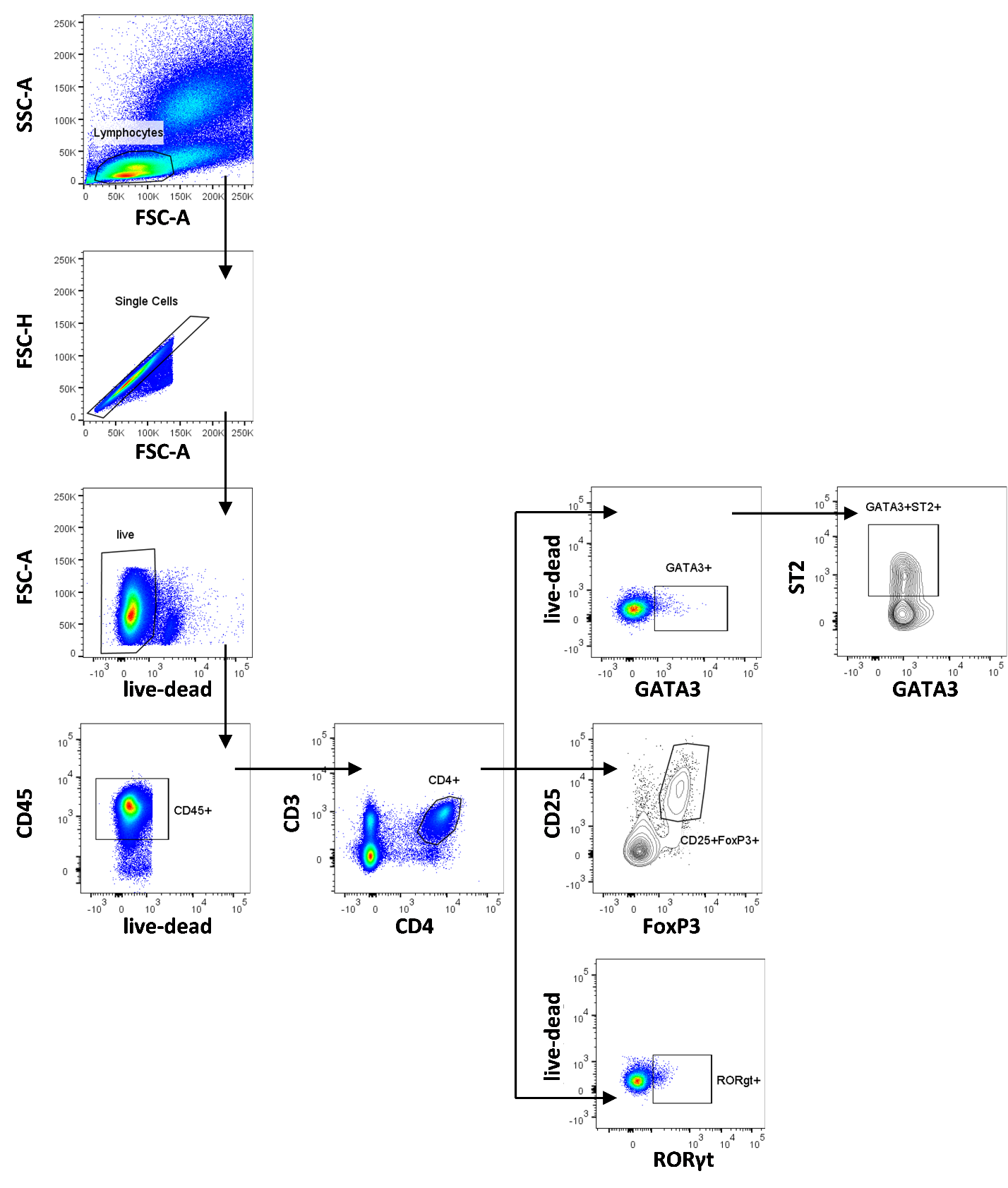


**Supplementary Figure 4.** Gating strategy for the characterization of T helper cell populations in pulmonary lymphocytes. Shown is the gating of one representative sample.

## Supplementary Tables

**Supplementary Table 1.** Antibodies for FACS analysis of BALF cells.

| **Marker** | **Fluorochrome** | **Clone** | **Manufacturer** |
| --- | --- | --- | --- |
| CD4 | AF700 | RM4-5 | BioLegend |
| CD8 | FITC | 53-6.7 | BD Biosciences |
| CD11b | BV711 | M1/70 | BioLegend |
| CD45 | PerCP-Cy5.5 | 30-F11 | BioLegend |
| CD206 | PE-Dazzle594 | C068C2 | BioLegend |
| F4/80 | APC-eF780 | BM8 | eBiosciences |
| Ly-6G | Pacific Blue | 1A8 | Biolegend |
| Siglec-F | PE | E50-2440 | BD Biosciences |
| Live/dead aqua fixable cell stain | 516 nm | - | Thermo Fisher |

**Supplementary Table 2.** Antibodies for FACS analysis of alveolar macrophages.

| **Marker** | **Fluorochrome** | **Clone** | **Manufacturer** |
| --- | --- | --- | --- |
| CD11c | BV785 | N418 | BioLegend |
| CD45 | Pacific Blue | 104 | BioLegend |
| CD11b | APC-e780 | M1/70 | eBiosciences |
| MHCII | AF700 | M5/114.15.2 | BioLegend |
| CD64 | APC | X54-5/7.1 | BioLegend |
| Ly6G | PE-Cy7 | 1A8 | BioLegend |
| SiglecF | PE | E50-2440 | BD Biosciences |
| 7-AAD | 647 nm | - | Thermo Fisher |

**Supplementary Table 3.** Antibodies for FACS analysis of Th cell subsets.

| Marker | Fluorochrome | Clone | Manufacturer |
| --- | --- | --- | --- |
| CD3ε | AF700 | 17A2 | BioLegend |
| CD4 | BV650 | RM4-5 | BioLegend |
| CD45 | APC-eF780 | 30-F11 | eBiosciences |
| ST2 | BV421 | DIH9 | BioLegend |
| CD25 | Biotin | 7D4 | eBiosciences |
| Streptavidin | PE-Cy7 | - | BioLegend |
| FoxP3 | PerCP-Cy5.5 | FJK-16s | eBiosciences |
| GATA3 | eF660 | TWAJ | eBiosciences |
| RORγt | PE | AFKJS-9 | eBiosciences |
| Live/dead aqua fixable cell stain | 405nm | - | Thermo Fisher |

**Supplementary Table 4.** Primers for analysis of *Cyp1a1* gene expression levels.

| **Primer** | **Sequence (5’ to 3’)** | **Manufacturer** |
| --- | --- | --- |
| CYP1A1_F | GACACAGTGATTGGCAGAG | Metabion International |
| CYP1A1_R | GAAGGTCTCCAGAATGAAGG | Metabion International |
